# *Pseudomonas aeruginosa-*secreted respiratory toxin HQNO triggers fatty acid accumulation in respiring *Staphylococcus aureus,* decreasing SaeRS-dependent transcriptional regulation

**DOI:** 10.1128/jb.00395-25

**Published:** 2025-10-08

**Authors:** Franklin Roman-Rodriguez, Nupur Tyagi, Hassan Al-Tameemi, Jeffrey M. Boyd

**Affiliations:** 1 Department of Biochemistry and Microbiology, Rutgers, The State University of New Jersey 242612, New Brunswick, New Jersey, USA; Geisel School of Medicine at Dartmouth, Hanover, New Hampshire, USA

**Keywords:** SaeRS, fatty acid, respiration, HQNO, *Pseudomonas aeruginosa*, *Staphylococcus aureus*

## Abstract

*Staphylococcus aureus* and *Pseudomonas aeruginosa* are the two pathogens that colonize the airway of cystic fibrosis patients. As patients age, *P. aeruginosa* outcompetes *S. aureus* to become the predominant organism in the airway, which overlaps with worsening symptoms. This inverse correlation is partly due to the ability of *P. aeruginosa* to secrete secondary metabolites and virulence factors that are antagonistic to the host cells and other bacteria present. Several of these secondary metabolites inhibit *S. aureus* respiration. SaeRS is a two-component regulatory system that promotes the transcription of numerous virulence genes in *S. aureus*. The transcription of SaeRS-regulated genes is decreased as a function of respiratory status. The accumulation of intracellular fatty acids also negatively impacts the activity of SaeRS. Incubation of *S. aureus* with *P. aeruginosa* cell-free conditioned culture medium decreased the transcriptional output of the SaeRS system. Further analyses using *P. aeruginosa* mutant strains and chemical genetics determined that 2-heptyl-4-quinolone N-oxide (HQNO) was responsible for the SaeRS-dependent changes in gene regulation. Treatment with HQNO increased the abundance of cell-associated fatty acids. HQNO inhibits cell respiration, and the SaeRS system did not respond to HQNO treatment in a respiration-impaired *S. aureus* strain, which accumulates fatty acids. The data presented are consistent with a working model wherein treatment of *S. aureus* with HQNO inhibits respiration, increasing free fatty acid accumulation, which negatively impacts SaeRS signaling. This results in decreased expression of the SaeRS regulon, which has significant roles in pathogenesis.

**IMPORTANCE:**

*Pseudomonas aeruginosa* and *Staphylococcus aureus* are often co-isolated from the airways of cystic fibrosis patients. *P. aeruginosa* secretes non-essential metabolites that alter *S. aureus* physiology, providing *P. aeruginosa* with a competitive advantage. *S. aureus* can adapt to the presence of these metabolites, but the genetic mechanisms used to sense these *P. aeruginosa*-produced metabolites and/or the induced physiological changes are largely unknown. The *S. aureus* SaeRS two-component regulatory system positively regulates the expression of various virulence factors, including toxins and proteases, that facilitate adaptation to and survival in hostile host environments. This study demonstrates that the *P. aeruginosa*-produced respiratory toxin 2-heptyl-4-quinolone N-oxide inhibits respiration, decreasing the transcription of SaeRS-regulated genes and thereby decreasing virulence factor production. These findings could be exploited to decrease the ability of *S. aureus* to express virulence factors in various infection settings.

## INTRODUCTION

*Staphylococcus aureus* is a commensal bacterium that can be found in the nares in approximately 30% of the human population (1). *S. aureus* typically causes mild skin infections but can also cause more severe and invasive diseases such as pneumonia, osteomyelitis, endocarditis, and septicemia (2–6). The use and misuse of antibiotics have increased the number of *S. aureus* isolates resistant to antibiotics, decreasing treatment strategies, which is a growing concern (7–10).

*S. aureus* is one of the primary bacteria that colonizes the airway and lungs of cystic fibrosis (CF) patients (11). While *Pseudomonas aeruginosa* can outcompete *S. aureus* in the laboratory and in the CF lung, it is believed that *P. aeruginosa* benefits from co-existence during infections (12, 13). *P. aeruginosa* secretes secondary metabolites that alter *S. aureus* physiology, including the inhibition of respiration (13–15). The 2-heptyl-4-quinolone N-oxide (HQNO) is a principal secondary metabolite produced by the expression of the *P. aeruginosa pqsABCDE* operon (16, 17). It has been hypothesized that HQNO blocks the oxidation and reduction of the cytochrome oxidases and the NADH dehydrogenase in the *S. aureus* respiratory chain (14, 15, 18). Respiratory inhibition results in *S. aureus* shifting to a fermentative metabolism to produce energy and balance redox (19, 20). This results in *S. aureus* secreting lactate, which is a preferred carbon source of *P. aeruginosa* (21).

*S. aureus* utilizes transcriptional regulatory systems, including two-component regulatory systems (TCRS), to sense and respond to environmental and internal stressors (22). TCRS are typically composed of a sensor kinase and a response regulator that has an altered affinity for DNA if phosphorylated. The *S. aureus* exoprotein regulator (Sae) TCRS controls the expression of numerous virulence factors (23–28). The Sae TCRS is composed of the membrane-spanning histidine kinase SaeS, which has autophosphorylation activity and serves as a phosphodonor for the response regulator SaeR (29, 30). Phosphorylation of SaeR positively regulates the transcription of several genes encoding virulence factors, including alpha-toxin (*hla*), leukocidins (*lukF*), proteases, and other immune evasion factors (29, 31, 32). Sae also utilizes two accessory proteins, the transmembrane protein SaeQ and the extracellular lipoprotein SaeP (29). SaePQ can interact with SaeS and activate its phosphatase activity toward SaeR. The transcription of the *saePQRS* operon is controlled by the *saeP1* promoter, which has two known binding sites for the SaeR response regulator (33, 34).

Although the direct stimuli for the SaePQRS system are unknown, increased free fatty acid levels negatively impact SaeRS-dependent transcription (35–37). We previously demonstrated that SaeRS-dependent regulatory output is altered during fermentative growth, suggesting that respiratory status alters SaeRS activity either directly or indirectly (38). Genetic inhibition of *S. aureus* respiration by the introduction of a mutation in the gene that codes for NADH oxidase (*ndhC*) decreased hemolysis activity. The inclusion of bovine serum albumin (BSA), which binds to and decreases the concentration of cell-accessible fatty acids, resulted in increased hemolysis (39). Both phenotypes depended on SaeRS, leading to the previously untested hypothesis that fatty acids accumulate upon respiration inhibition and cause decreased SaeRS activity (39).

This manuscript tests the hypothesis that one or more *P. aeruginosa*-excreted secondary metabolites inhibit *S. aureus* respiration, resulting in fatty acid accumulation and decreased SaeRS-dependent transcription. In a companion manuscript, we demonstrate that *P. aeruginosa*-secreted HQNO inhibits *S. aureus* respiration under the growth conditions utilized herein. In this manuscript, we demonstrate that inhibition of respiration increases fatty acid accumulation, which results in decreased SaeRS-dependent transcription. These findings increase our knowledge of *S. aureus* and *P. aeruginosa* interactions, and they also suggest that HQNO could be used to decrease SaeRS-dependent gene regulation, which is important for expressing and secreting numerous *S. aureus* virulence factors.

## MATERIALS AND METHODS

### Bacterial strains and culture conditions

Tryptic soy broth (TSB) was purchased from VWR. TSB was supplemented with 1.5% agar (VWR) to generate a solid tryptic soy agar (TSA) medium. Unless stated otherwise, bacterial strains were grown aerobically in 2 mL of TSB in 10 mL culture tubes and shaken at 200 rpm at 37°C with a 30 degree angle to promote dioxygen respiration. HQNO (Selleck Chemicals) was prepared as a 5 mg mL^-1^ stock in dimethyl sulfoxide (DMSO). To assess the impact of HQNO, strains were cultured for 8 hours at 37°C with or without 5 µg mL^-1^ HQNO, oleic acid (0.005% wt/vol), and/or BSA (10 µg mL^-1^). To examine the possibility of BSA binding to HQNO, solutions prepared in TSB media were filtered through a Centricon-10 (10,000 MW cutoff) (Amicon) for 15 min at 5,000 × *g* before adding to the strains containing the *saeP1* transcriptional reporter. When selecting for plasmids or chromosomal insertions, antibiotics were added to a final concentration of 30 µg mL^−1^ chloramphenicol (Cm), 10 µg mL^−1^ erythromycin (Erm), or 5 µg mL^−1^ tetracycline (Tet). A total of 10 µg mL^−1^ chloramphenicol was used to maintain plasmids.

### Bacterial strains and plasmids

All plasmids and strains utilized for this study are listed in Table 1. Bacteriophage 80α was used to perform the transductions (40). Bacterial strains and plasmids were PCR-verified or sequence-verified by Azenta (South Plainfield, NJ). DNA primers were purchased through Integrated DNA Technologies (Coralville, IA) (Table 2). To create the pOS_*hla_gfp* transcriptional reporter plasmid, Phusion polymerase (New England Biolabs) was used to amplify the *hla* promoter using the following primer pair: hla5hindIII and hla3kpnI. PCR products were gel-purified (Qiagen) and subsequently digested and ligated into similarly digested pOS*_saeP1_gfp* (41) using Quick Ligase (New England Biolabs). The ligation product was transformed into *Escherichia coli* DH5-α cells (NEB) and selected for growth in lysogeny broth (LB) agar plates containing 100 µg mL^−1^ ampicillin. Clones were verified by PCR followed by sequence verification.

**TABLE 1:** Strains and plasmids used in this study

Strain	Genotype	Source
*Pseudomonas aeruginosa* PA14 strains		
JMB 10389	PA14	(42)
JMB 10391	Δ*phz*	(43)
JMB 10393	Δ*pqsABC*	(44)
JMB 10394	Δ*hcn*	(45)
JMB 13827	Δ*pqsL*	(45)
JMB 13828	Δ*pqsH*	(45)
*Staphylococcus aureus* LAC strains		
JMB 1100	USA300_LAC	(46)
JMB 1874	*hla:Tn*(*ermB*)	(47) This study
JMB 2049	*ndh2b::tetM* (SAUSA300_0841)	This study
JMB 2970	*ndh2a::Tn(ermB)* (SAUSA300_0844)	This study
JMB 15557	*ndh2a::Tn(ermB) ndh2b::tetM*	(47) This study
JMB 3900	*saeP::Tn*(*ermB*)	This study
JMB 7100	*saeR::Tn*(*ermB*)	(38)
JMB 8988	Δ*cydA qoxB::tet*	This study
JMB 13703	Δ*cydA qoxB::tet saeR::Tn*(*ermB*)	This study
JMB 1432	*fur::tet*	(48)
JLB331	Δ*saeRS*	(49)
JLB137	Δ*saeQ*	(49)
JLB140	Δ*saeP*	(49)
JLB155	*P_xyl/tetO_-sae*	(49)
JLB304	*∆saePQ P_xyl/tetO_-sae*	(49)

Plasmid	Purpose	Source
pOS_*saeP1_gfp*	*saeP1* transcriptional reporter	(41)
pOS_*hla_gfp*	*hla* transcriptional reporter	This study
pJB38_Δ*cyd*	Generate Δ*cyd* mutant	This study
pJB38_Δ*qox::tet*	Generate Δ*qox::tet* mutant	This study
pJB38_Δ*ndh2b*	Generate Δ*ndh2b::tet* mutant	This study
pJB38_Δ*ndh2b::tet*	Generate Δ*ndh2b::tet* mutant	This study

**TABLE 2:** Primers used for this study

Name	Sequence
Ycc forward	AATAGGCGTATCACGAGGCCCTTTCGTCTTCAAGAATTCGGTGGCACTTTTCGGGGAAA
YCC qoxB rev	TGATGGCATTATGGTGCATCTTACGCTAGCGCACATTAGGACCGTTATAGTTACGCTAT
YCC qoxB up for	ATAGCGTAACTATAACGGTCCTAATGTGCGCTAGCGTAAGATGCACCATAATGCCATCA
qoxB tetR up rev	CTTCATCATCGGTCATAAAATCCGACGCGTTACCTTTAACTAGTAATTGATCCCATGG
qoxB tetR for	CCATGGGATCAATTACTAGTTAAAGGTAACGCGTCGGATTTTATGACCGATGATGAAG
tetR ycc rev qoxB	GCCTCCCTTTCTTTAATACGCGCTTCACGCGTTTAGAAATCCCTTTGAGAATGTTTATA
tetR qoxB dwn for	TATAAACATTCTCAAAGGGATTTCTAAACGCGTGAAGCGCGTATTAAAGAAAGGGAGGC
tetR pJB38 rev dwn qoxB	CTAGAGGATCCCCGGGTACCGAGCTCGAATTCGTGAAGAGTGACCGCCTTGCATAACCC
0841upEcoR1	GGGGAATTCCCCAGTTATGTAATAGTGCCTTAGTTAGTAC
0841dwnSal1	GGGGTCGACGCAAGTTGCGTTAATGCGCCAACATCG
0844 BamH1	CCCGGATCCGGCTGTGGTAGTTCATTTAGAACTGC
0844SaI1	CCCGTCGACGCCCTTCAGTGACACTGAAGGAC
Ycc forward	AATAGGCGTATCACGAGGCCCTTTCGTCTTCAAGAATTCGGTGGCACTTTTCGGGGAAA
YCC CDS rev	TCTTCAACTCAAAACCTGCAGGCGGCTAGCGCACATTAGGACCGTTATAGTTACGCTAT
YCC CDS up for	ATAGCGTAACTATAACGGTCCTAATGTGCGCTAGCCGCCTGCAGGTTTTGAGTTGAAGA
CDS up rev	TATGATTTCTTTCCTTCAACATATTCACGCGTACCATAACACTGTTATACCTATAAATG
CDS dwn for	CATTTATAGGTATAACAGTGTTATGGTACGCGTGAATATGTTGAAGGAAAGAAATCATA
pJB38 dwn rev CDS	CTAGAGGATCCCCGGGTACCGAGCTCGAATTCAGAAATTGGATTCCCAATTTCTACAGA
GFP verification	GTGTCCATTTACATCTCCGTCAAGTTC
RT-hla-F	TCGTTCAAGGTCCCGATTTT
RT-hla-R	CGGTGGGTTTGTATAATTATTGCTT
*saeR* For	AAGAACATGATACCATTTACGCCTTA
*saeR* Rev	CCCTTGGACTAAATGGTTTTTTGA
hla3kpnI	GGGGGTACCCCTTCTATTTTTTAAAACGATTTGAGGAAACAATAATC
hla5hindIII	GGGAATCTTGGGCATTTTCATTCGGTCAACTACTACC
G+tet mluI	CCCACGCGTTTAGAAATCCCTTTGAGAATGTTT
G+tet nheI	CCCGCTAGCCGGATTTTATGACCGATGATGAAG
0841upEcoRI	GGGGAATTCCCCAGTTATGTAATAGTGCCTTAGTTAGTAC
0841UpfuseNheI	ACGCGTGGTACCGCTAGCGCTAGCCTGTATTCACTTGTGGATGATTAGGG
0841dwnfusMluI	GCTAGCGGTACCACGCGTACGCGTGCCGAAGTTCAAGGTGATCAAATTGCCG
0841dwnSalI	GGGGTCGACGCAAGTTGCGTTAATGCGCCAACATCG

The pJB38_Δ*cyd* and pJB38_Δ*qox::tet* plasmids were made using yeast recombinational cloning (50, 51). To generate the pJB38_Δ*qox::tet* vector, the yeast cloning cassette was amplified using pJB38_Δ*copAZ* (52) with the following primer pair: Ycc forward and YCC qoxB rev. The upstream and downstream regions of *qox* were amplified using the following primer pairs: YCC qoxB up for and qoxB tetR up rev; tetR qoxB dwn for and tetR pJB38 rev dwn qoxB. The *tet* gene was amplified using *fur::tet* (JMB1432 [53]) chromosomal DNA as a template and the following primer pair: qoxB tetR for and tetR ycc rev qoxB. The mutant was generated by allelic replacement as previously described (54). To generate the pJB38_Δ*cyd,* the yeast cloning cassette was amplified from pJB38_Δ*copAZ* using the following primer pair: Ycc forward and YCC CDS rev. The DNA regions upstream and downstream of *cyd* were amplified using the following primer pairs: YCC CDS up for and CDS up rev; CDS dwn for and pJB38 dwn rev CDS. Clones were verified by PCR followed by sequence verification.

The pJB38_Δ*ndh2b::tet* plasmid was made as previously described (55). The *ndh2b* upstream and downstream regions were amplified using the following primer pairs: 0841upEcoRI and 0841UpfuseNheI; 0841dwnfusMluI and 0841dwnSalI. The amplicons were then used as template for a second PCR reaction using the 0841upEcoRI and 0841dwnSalI primers. The amplicon was digested with EcoRI and SalI and ligated into similarly digested pJB38, creating pJB38_Δ*ndh2b*. The *tetM* allele was amplified using cells from strain JMB1432 as a template and the following primer pair: G+tet nheI and G+tet mluI. The amplicon was digested with NheI and MluI and ligated into pJB38_Δ*ndh2b* that had been similarly digested, creating pJB38_Δ*ndh2b::tet*

### Transcriptional reporter assays

*S. aureus* strains containing transcriptional reporters were grown overnight in TSB containing 10 µg mL^-1^ Cm and subsequently diluted to a 0.1 (A_600_) in 2 mL of TSB-Cm in 10 mL culture tubes in triplicate. Strains that had *saeRS* under the transcriptional control of *tetRO xylR* were supplemented with 2% (wt/vol) xylose. After 8 hours of growth, 200 µL from each sample was added to black 96-well plates (Thermo Scientific), and fluorescence emission was measured at a 488 nm excitation, 510 nm emission, and 12 nm path length using a Varioskan Lux plate reader (Thermo Scientific). HQNO (5 µg mL^-1^) and/or oleic acid (0.005%) and/or BSA (10 µg mL^-1^) were added to samples and incubated for 8 hours before quantifying Gfp fluorescence in all cultures.

To generate *P. aeruginosa* conditioned media, strains were grown overnight in 3 mL of lysogeny (Luria) broth(LB) broth in a 30 mL culture tube. The overnight culture was centrifuged to pellet cells, and the supernatant was decanted, followed by filter sterilization. A total of 100 µL of the *P. aeruginosa* cell-free conditioned media was added to 2 mL *S*. *aureus* cultures.

For assays with fatty acid addition, the overnight cultures were diluted in triplicate to an optical density (A_600_) of 0.1 in 2 mL of TSB with 10 µg mL^−1^ Cm in 10 mL culture tubes. Cells were grown until they reached an optical density (A_600_) of one, and three sets of triplicate samples were prepared: one with HQNO at 5 µg mL^−1^, one with 0.005% oleic acid, and one containing both HQNO and oleic acid at the concentrations previously mentioned. Cells were grown for an additional 2 hours at 37°C with constant shaking at 200 rpm before measuring fluorescence. Sample fluorescence was standardized to individual culture optical density (A_600_) at the time of assay for all transcriptional reporter assays.

### Hemolysis assays

Bacterial cultures were grown overnight in TSB with and without 5 µg mL^−1^ of HQNO in triplicate. Strains were cultured overnight, followed by standardizing the cultures to an optical density (A_600_) of 0.1 in 1 mL of phosphate buffered saline (PBS). This PBS/conditioned-medium combination was centrifuged at 13,000 × *g* for 1 min, and the supernatant was removed and filter sterilized. A 1% solution of defibrinated rabbit blood cells (Hemostat) was prepared by taking 15 µL of blood and centrifuging at 1,700 × *g* for 5 min. The supernatant was removed, and the red blood cells were carefully resuspended in 1 mL of PBS. The washing and resuspending steps were repeated up to three times, and the red blood cells were finally resuspended in 1.5 mL of PBS. The experiments were conducted using a 96-well plate by adding 50 µL of the red blood cell solution and 50 µL of the cell-free culture supernatant. As previously reported, optical density (A_600_) was measured every 15 min for 2 hours (56). The linear portion of the lysis curve, containing at least four data points, was fitted to a best-fit line, and lysis rates were reported as the decrease in absorbance per minute.

### Quantification of intracellular fatty acids

Bacterial strains were cultured overnight in TSB and diluted in triplicate to an OD_600_ of 1 in 5 mL TSB with or without 5 µg mL^−1^ HQNO in 30 mL glass culture tubes. Culture density was recorded after 12 hours of growth, and cells were placed on ice. The equivalent of 10 optical density units (A_600_) of cells was transferred to 15 mL conical centrifuge tubes, and the cells were pelleted by centrifugation (Eppendorf 5810R). Cell pellets were resuspended in 1.5 mL of PBS and transferred to 2 mL centrifuge tubes. Cells were pelleted by centrifugation at 13,000 × *g* for 1 min and resuspended in 300 µL of a 1% (vol/vol) Triton X-100 chloroform solution in 2 mL screw cap tubes containing 0.1 mm silica glass beads (MP Biomedicals). Cells were lysed by bead beating (two cycles, 40 s each, 6.0 m/s) using a FastPrep homogenizer (MP Biomedicals). The cellular material was centrifuged at 13,000 × *g* for 10 min to remove insoluble debris. Equal amounts of the organic phase from each sample were collected and transferred to a 1.5 mL centrifuge tube. The lipids were air-dried at 50°C for 1 hour in a fume hood to remove chloroform. The dried lipids were dissolved in 100 µL of assay buffer and vortexed extensively for 5 min. The concentration of free fatty acids from each sample was determined fluorometrically using a free fatty acid quantitation kit (Sigma-Aldrich). Fluorescence measurements were taken at room temperature using a black 96-well plate on a Varioskan Lux microplate reader (Thermo Scientific) with an excitation wavelength of 535 nm, an emission wavelength of 587 nm, and a 12 nm path length.

## RESULTS

### *P. aeruginosa* conditioned media alters SaeRS-dependent transcriptional activity

We previously demonstrated that the absence of a terminal electron acceptor alters SaeRS regulatory output (38). *P. aeruginosa* secretes secondary metabolites that inhibit dioxygen respiration in *S. aureus* (14, 15, 18, 57, 58). We tested the hypothesis that secondary metabolites secreted by *P. aeruginosa* alter SaeRS-dependent regulatory activity. The *S. aureus hla* codes for α-hemolysin, a virulence factor, and cytotoxin directly regulated by SaeR (59). To monitor *hla* transcriptional activity, we placed *gfp* under the transcriptional control of the *hla* promoter, allowing us to measure fluorescence as an indicator of *hla* transcriptional output.

**Fig 1:**
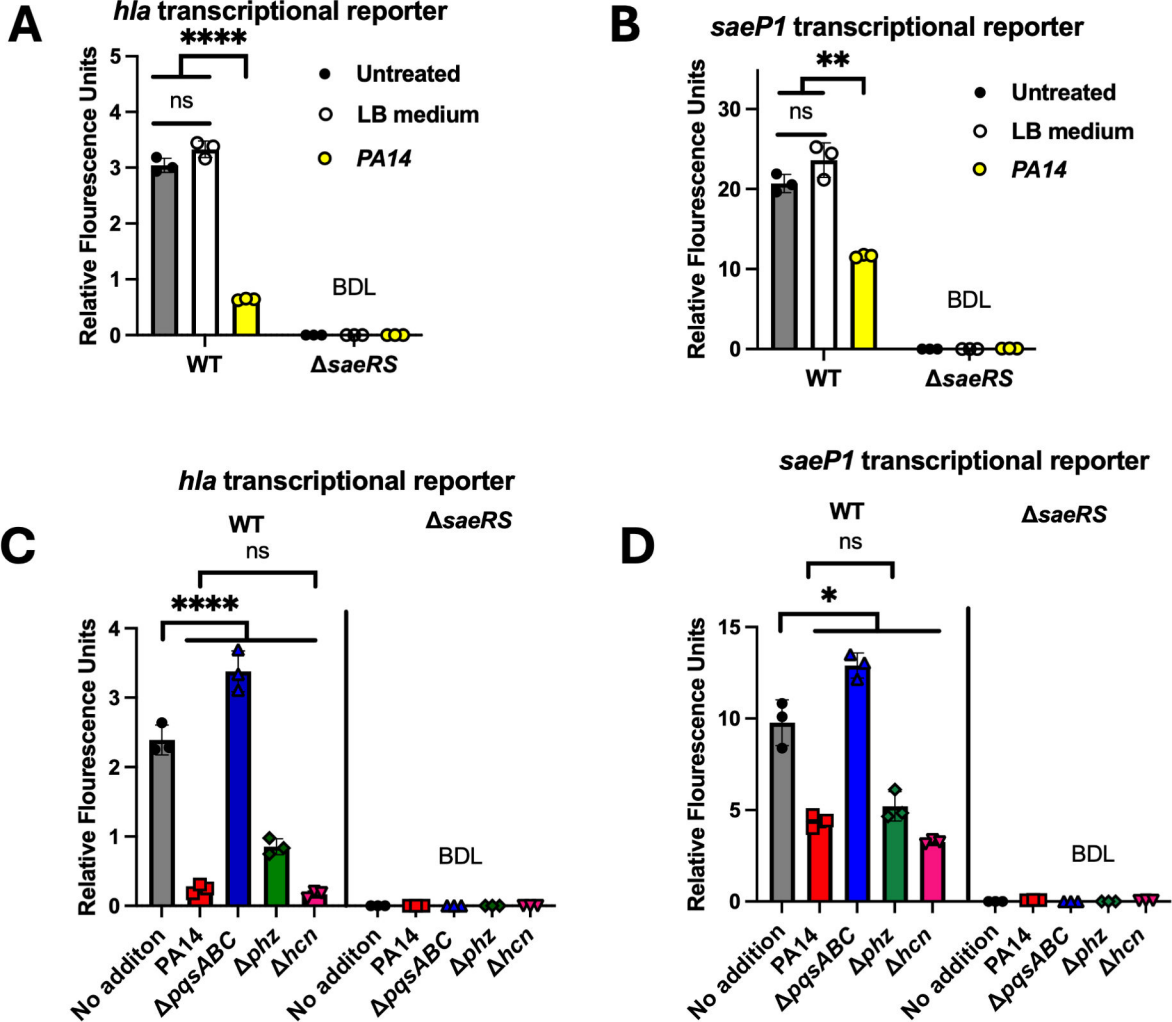
*P. aeruginosa* cell-free conditioned culture media decreases SaeRS-dependent transcriptional activity. (**A and B**) The expression of *gfp* was quantified in wild-type *S. aureus* (WT; JMB1100) and the isogenic Δ*saeRS* (JLB 331) mutant carrying either the pOS_*hla_gfp* (A) or pOS_*saeP1_gfp* (B) transcriptional reporters after culture in TSB-Cm with or without cell-free *P. aeruginosa* conditioned culture medium. (**C and D**) The expression of Gfp was quantified in the WT and Δ*saeRS* strains carrying the pOS_*hla_gfp* (C) or pOS_*saeP1_gfp* (D) transcriptional reporters after culture in TSB-Cm with or without cell-free conditioned culture media isolated from isogenic *P. aeruginosa* strains. Each individual data bar corresponds to the average of the biological replicates (*n* = 3), and standard deviations are shown, but the data points sometimes obscure them. Student’s unpaired *t*-tests were performed on the data displayed in A and B. The data presented in C and D were analyzed using a one-way analysis of variance, and *ad hoc* Tukey tests were conducted for pairwise comparisons. The * denotes a *P*-value ≤0.05 and **** ≤0.0001, and ns denotes no significant difference. BDL denotes below the detectable limit of the fluorimeter.

*P. aeruginosa* PA14 (PA14) was cultured in LB culture medium, and the conditioned medium was harvested, sterilized, and retained. The cell-free conditioned LB culture medium was added to liquid cultures of *S. aureus* USA300_LAC (wild type or WT), and the isogenic Δ*saeRS* mutant containing a *hla* transcriptional reporter and fluorescence was quantified. There was a significant decrease in *hla* transcriptional activity upon co-culture with the PA14 conditioned medium (Fig. 1A). The transcriptional activity of *hla* was below the detectable limit in the Δ*saeRS* mutant, confirming that *hla* transcription requires SaeR. The transcriptional output of the WT strain after the addition of LB medium phenocopied the no-addition control, indicating that *S. aureus* responds to one or more PA14-produced metabolites (data not shown).

The *S. aureus saeP1* operator controls the transcriptional activity of the *saePQRS* operon. Phosphorylated SaeR binds to two sites in the *saeP1* operator to induce transcription of *saePQRS* (33, 60). Using a fluorescent transcriptional reporter, we quantified *saeP1* transcriptional activity in the WT and Δ*saeRS* mutant after adding PA14 conditioned culture medium. The added conditioned medium significantly decreased *saeP1* transcriptional activity in the WT strain (Fig. 1B). The Δ*saeRS* mutant has no detectable transcriptional activity, confirming the dependence on SaeR for transcription. These findings are consistent with the hypothesis that one or more PA14-secreted metabolites can decrease the activity of SaeRS.

### The respiratory toxin HQNO stimulates SaeRS

We sought to determine which PA14 secondary metabolites from the conditioned culture medium were altering SaeRS signaling. We harvested conditioned culture media from isogenic *P. aeruginosa* mutants deficient in producing one or more secondary metabolites. Conditioned media was isolated from PA14 cultures and strains deficient in synthesizing phenazines (Δ*phz*), hydrogen cyanide (Δ*hcn*), or quinolones (Δ*pqsABC*). This conditioned media was then added to WT and Δ*saeRS* cultures carrying either the *hla* or *saeP1* transcriptional reporters. While the conditioned culture medium from PA14 decreased *hla* and *saeP1* transcriptional activities, the conditioned medium from the Δ*pqsABC* mutant did not (Fig. 1C and D). The conditioned media from Δ*phz* and Δ*hcn* strains significantly decreased transcriptional activity to levels similar to the cultures containing the PA14 medium. The transcriptional activities of *hla* and *saeP1* were below the detectable limit in the Δ*saeRS* mutant. These data reinforce the hypothesis that the PQS system produces one or more molecules that decrease SaeRS-dependent transcriptional regulation.

**Fig 2:**
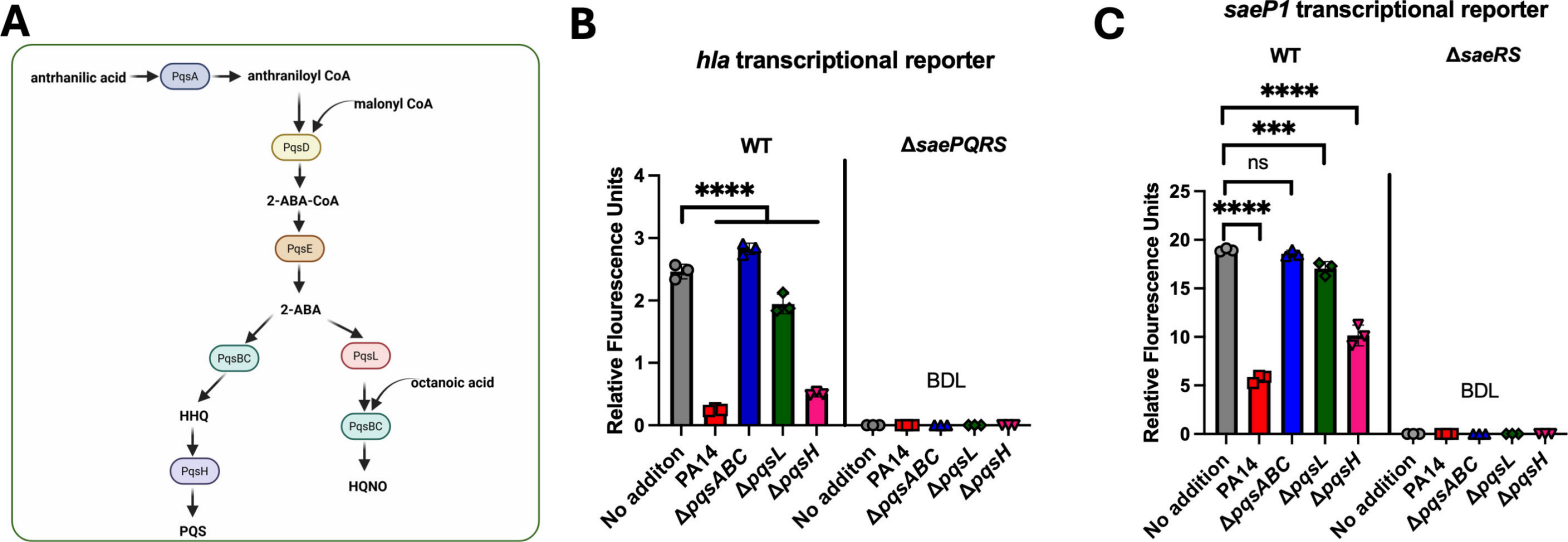
PqsABC or PqsL is required to alter SaeRS-dependent transcriptional activity. (**A**) Overview of the *P. aeruginosa* PQS system and its products. The gene product names and metabolite abbreviations are displayed. (**B and C**) Gfp expression was quantified in the *S. aureus* WT (JMB 1100) and isogenic Δ*saeRS* (JLB 331) mutant harboring either the pOS*_hla_gfp* (B) or pOS*_saeP1_gfp* (C) transcriptional reporter plasmids after the addition of cell-free conditioned media from the indicated isogenic *P. aeruginosa* strains. Each individual data bar corresponds to the average of the biological replicates (*n* = 3), and standard deviations are shown, but the data points sometimes obscure them. One-way analyses of variance were performed for all data sets, and *ad hoc* Tukey tests were conducted for pairwise comparisons. The *** denotes a *P*-value ≤0.001 and **** ≤0.0001, and ns denotes no significant difference. BDL denotes below the detectable limit of the fluorimeter.

We sought to identify which secondary metabolites generated from anthranilic acid altered Sae activity. In the PQS system, the *pqsL* and *pqsH* gene products are responsible for the production of HQNO and PQS, respectively (Fig. 2A) (61–63). Conditioned media from PA14, Δ*pqsL,* or Δ*pqsH* mutants significantly decreased *hla* transcriptional activity to varying degrees, whereas conditioned medium from the Δ*pqsABC* mutant did not (Fig. 2B). Conditioned medium from the Δ*pqsL* mutant had a diminished ability to decrease *hla* transcription when compared to the media isolated from PA14 or Δ*pqsH*.

Conditioned culture medium from the Δ*pqsL* mutant did not alter *saeP1* transcriptional activity, while conditioned culture medium from the Δ*pqsH* mutant did, but to a lower level than that of the Δ*pqsABC* mutant (Fig. 2C). These results led to a model wherein the PqsL-produced HQNO alters SaeRS transcriptional output, which requires the presence of *saeRS*.

**Fig 3:**
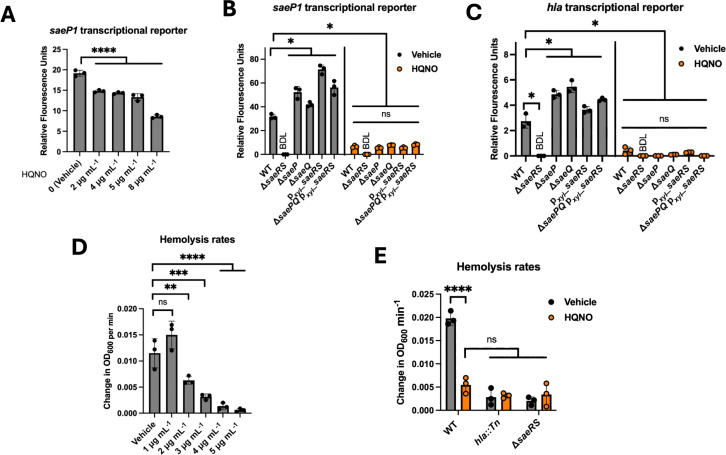
HQNO alters SaeRS-dependent gene expression. (**A**) The *S. aureus* WT (JMB 1100) containing the pOS*_saeP1_gfp* transcriptional reporter plasmid was challenged with various concentrations of HQNO, and Gfp expression was quantified. (**B and C**) Gfp expression was quantified in WT, Δ*saeP* (JLB 140), Δ*saeQ* (JLB 137), Δ*saeRS* (JLB 331), P_xyl__*saeRS* (JLB 155), and *∆saePQ* P_xyl__*saeRS* (JLB 304) strains harboring either the pOS*_saeP1_gfp* (B) or pOS*_hla_gfp* (C) transcriptional reporter plasmids after challenge with or without HQNO (5 µg mL^−1^). Strains JLB 155 and 304 were cultured with 2% (wt/vol) xylose. (**D**) WT *S. aureus* was challenged with various concentrations of HQNO, and the conditioned culture media was isolated. The conditioned media was then added to a solution of 1% rabbit red blood cells, and hemolysis was monitored over time spectrophotometrically. (**E**) A cell-free conditioned culture medium was isolated from the WT, *hla::Tn* (JMB 1874), and Δ*saeRS* (JLB 331) strains after challenge with 5 µg mL^−1^ HQNO. Rabbit red blood cell hemolysis was monitored over time, and hemolysis rates were quantified. Each individual data bar corresponds to the average of the biological replicates (*n* = 3), and standard deviations are shown, but the data points sometimes obscure them. The data in A and D were analyzed using one-way analysis of variance (ANOVA), and *ad hoc* Tukey tests were conducted for pairwise comparisons. The data in B, C, and E were analyzed using two-way ANOVA, and *ad hoc* Tukey tests were conducted for pairwise comparisons. The * denotes a *P*-value ≤0.05, ** ≤0.01, *** ≤0.001, and **** ≤0.0001, and ns denotes no significant difference. BDL denotes below the detectable limit of the fluorimeter.

We began testing the hypothesis that HQNO alters SaeRS-dependent transcriptional activity. Titrating HQNO into the WT strain containing the *saeP1* transcriptional reporter resulted in a dose-dependent decrease of *saeP1* transcriptional activity (Fig. 3A). HQNO was dissolved in DMSO (vehicle control), and the addition of up to 1% DMSO did not alter growth kinetics or *saeP1* transcriptional activity (data not shown).

SaeP and SaeQ interact with SaeS and promote phosphatase activity (29). We decided to test whether the HQNO-dependent decrease in *saeP1* transcriptional activity was altered in strains lacking SaeP or SaeQ (Δ*saeP* and Δ*saeQ*). Additionally, we tested if the HQNO effect required DNA binding activity (Δ*saeRS*). The Δ*saeP* and Δ*saeQ* strains had increased *saeP1* transcription, suggesting more phosphorylated SaeR (Fig. 3B). The fluorescence in the Δ*saeRS* mutant was very low, demonstrating that transcription of *saeP* requires the SaeR DNA-binding protein. All strains tested had decreased transcriptional activity upon growth in the presence of HQNO.

We examined whether the alterations in SaeRS activity required *saeRS* to be under the transcriptional control of the *saeP1* promoter (49). To this end, we examined the effect of HQNO on SaeRS activity in a strain with *saePQRS* under the transcriptional control of a xylose-inducible promoter (p*_xyl/tetO_-sae*). We utilized strains that contained *saePQ* or did not. The basal level of *saeP1* transcriptional activity was slightly higher in the p*_xyl/tetO_-sae* strain compared to the WT strain (Fig. 3B). Upon growth with HQNO, the transcriptional activity of *saeP1* decreased in the p*_xyl/tetO_-sae* strain to a level that was equivalent to that of the WT. This phenotype occurred irrespective of the presence of SaePQ.

We also examined the effect of HQNO on *hla* promoter activity in the WT, Δ*saeP,* Δ*saeQ,* Δ*saeRS*, and p*xyl*_*saeRS* strains. In the absence of HQNO, the transcriptional activity of *hla* was increased in the Δ*saeP* and Δ*saeQ* mutant strains compared to the WT (Fig. 3C). The transcriptional activity of *hla* required the presence of SaeRS. The transcriptional activity was slightly increased in the strains containing *saeRS* under the transcriptional control of the xylose-inducible promoter. Growth with HQNO decreased transcription in all strains examined. The data in Fig. 3B and C suggest that the changes in SaeRS activity caused by the addition of HQNO are not the result of *saeRS* transcriptional changes, but rather altered stimulation of SaeRS.

We next examined the effect of HQNO on *hla* expression. We cultured the WT, *hla::Tn*, and Δ*saeRS* strains in the presence and absence of HQNO, isolated the conditioned culture media, and then quantified the ability of the conditioned media to lyse red blood cells, which correlates with the quantity of Hla present (56). The rate of hemolysis decreased in assays using the WT conditioned media as a function of increasing concentrations of HQNO that was included in the co-culture (Fig. 3D). Very little hemolysis was noted using conditioned culture media isolated from *hla::Tn* or Δ*saeRS* strains, which is consistent with published findings (37, 56). The rate of hemolysis catalyzed by cell lysates from these strains was not altered upon co-culture with HQNO (Fig. 3E). These results demonstrate that HQNO produced by PA14 decreased SaeRS-dependent transcriptional output.

### Active respiration is required for HQNO to negatively impact SaeRS-dependent transcription

**Fig 4:**
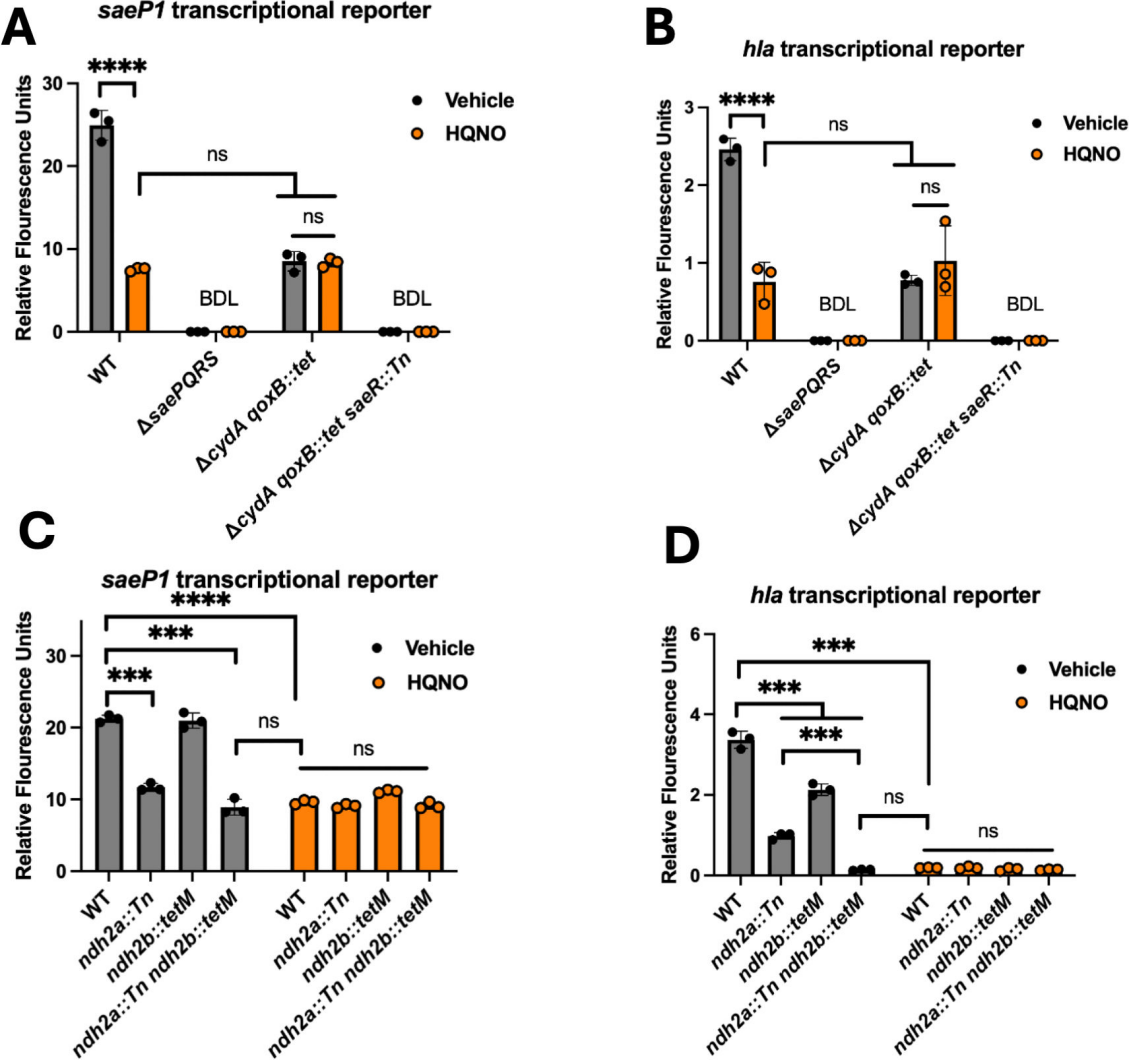
*S. aureus* must be respiring for HQNO to alter SaeRS-dependent transcription. (**A and B**) The expression of Gfp was quantified in the *S. aureus* WT (JMB 1100), Δ*saeRS* (JLB 331), Δ*cydA qoxB::Tn* (JMB 8989), and Δ*cydA qoxB::Tn saeR::Tn* (JMB 13703) strains containing either the pOS*_saeP1_gfp* (A) or pOS*_hla_gfp* (B) transcriptional reporter plasmids cultured with or without 5 µg mL^−1^ HQNO. (**C and D**) Gfp expression was quantified in WT *S. aureus*, *ndh2A::Tn* (JMB 2970), *ndh2b::tetM* (JMB 2049), and *ndh2A::Tn ndh2b::tetM* (JMB 15557) containing either the pOS*_saeP1_gfp* (C) or pOS*_hla_gfp* (D) transcriptional reporters cultured with or without 5 µg mL^−1^ HQNO. Each individual data bar corresponds to the average of the biological replicates (*n* = 3), and standard deviations are shown, but the data points sometimes obscure them. The data were analyzed using two-way analysis of variance, and *ad hoc* Tukey tests were conducted for pairwise comparisons. For both data sets, **** denotes a *P*-value ≤0.0001, and ns denotes no significant difference. BDL denotes below the detectable limit of the fluorimeter.

In a companion manuscript, we demonstrate that *S. aureus* has lower membrane potential and a reduced rate of dioxygen consumption when challenged with HQNO, suggesting that HQNO inhibits respiration under the growth conditions utilized (64). We tested the hypothesis that cells must be actively respiring for HQNO treatment to affect SaeRS-dependent transcription. The *S. aureus* genome codes for the Cyd and Qox terminal oxidases (65). We cultured the WT and Δ*cydA qoxB::Tn* double mutant strains containing the *saeP1* or *hla* transcriptional reporters in the presence and absence of HQNO and quantified transcriptional activity. The activities of both promoters were significantly decreased in the Δ*cydA qoxB::Tn* strain compared to the WT (Fig. 4A and B). The transcriptional activities in the Δ*cydA qoxB::Tn* strain phenocopied the transcriptional activities of the WT strain co-cultured with HQNO. The transcription of *saeP1* and *hla* was not significantly affected by HQNO in the Δ*cydA qoxB::Tn* strain. These data are consistent with the hypothesis that *S. aureus* must be actively respiring for HQNO to impact SaeRS transcriptional output negatively. It is also consistent with our previous findings demonstrating that respiration alters SaeRS output (38).

We wanted to test whether HQNO altered *saeP1* and *hla* transcriptional activity in strains lacking one or both NADH-dehydrogenases (*ndh2a, ndh2b*). A mutation in *ndh2a,* but not *ndh2b,* reduced *saeP1* transcriptional activity compared to the WT (Fig. 4C). The *ndh2a::Tn ndh2b::tetM* double mutant had a similar effect to the *ndh2a::Tn* single mutant, suggesting that *ndh2a* is the primary NADH dehydrogenase under the growth conditions utilized. Treatment of the single and double *ndh* mutant strains phenocopied the HQNO-treated WT strain. We repeated these assays using the *hla* transcriptional reporter (Fig. 4D). Both *ndh2a::Tn* and *ndh2b::tetM* alleles caused a significant decrease in *hla* transcriptional activity when compared to WT, with *ndh2a::Tn* showing the stronger phenotype. The *ndh2a::Tn ndh2b::tetM* alleles had an additive effect in reducing *hla* transcriptional activity. Treatment of the single and double *ndh* mutant strains phenocopied the *hla* transcriptional output of the treated WT strain. These data confirm that SaeRS transcriptional activity is impacted by respiratory status and demonstrate that active respiration is required for HQNO treatment to impact SaeRS signaling.

### Treatment with HQNO increases fatty acid accumulation

**Fig 5:**
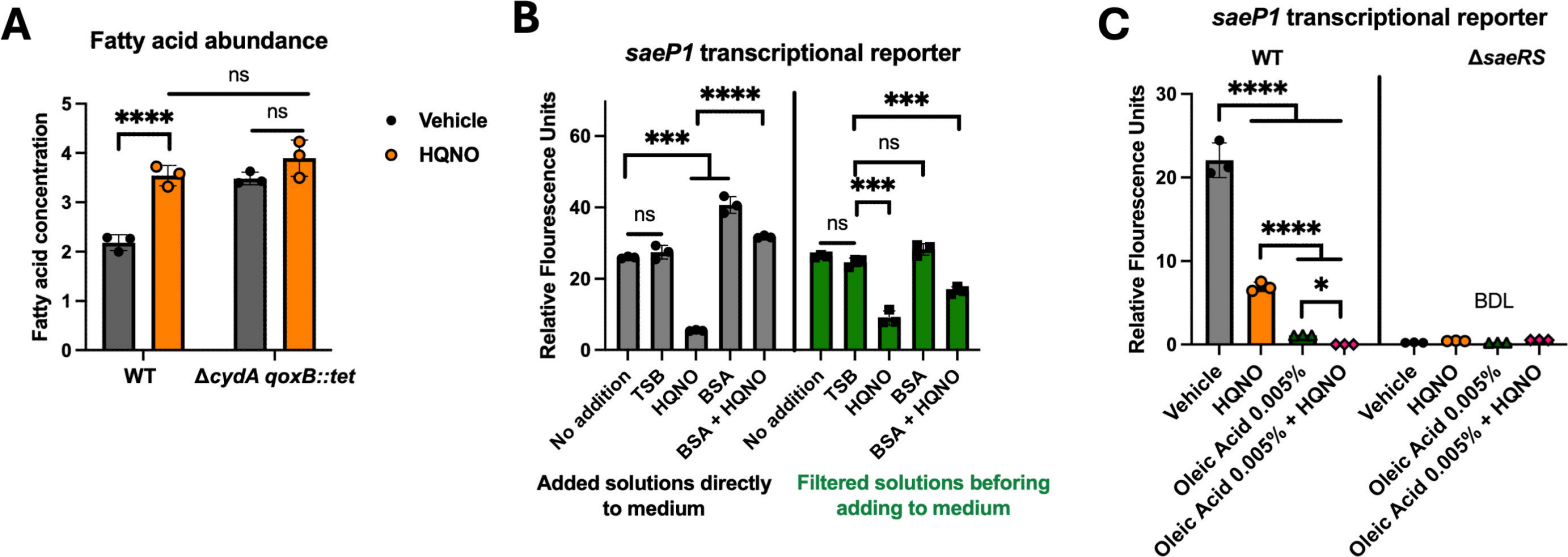
HQNO increases free fatty acid accumulation in *S. aureus*. (**A**) Free fatty acid concentrations were quantified in the *S. aureus* WT (JMB1100) and Δ*cydA qoxB::Tn* (JMB 8989) strains after culture with or without 5 µg mL^−1^ HQNO. (**B**) The WT harboring the pOS*_saeP1_gfp* transcriptional reporter plasmid was cultured with the following additions for 8 hours before reading fluorescence: TSB, 5 µg mL^−1^ HQNO, BSA (10 µg mL^−1^), and HQNO (5 µg mL^−1^) plus BSA (10 µg mL^−1^). For the samples on the right side of the side of the panel, the compounds were filtered through a 10,000 molecular weight cutoff filter to remove BSA before adding to the cultures. (**C**) The WT (JMB 1100) harboring the pOS*_saeP1_gfp* plasmid was treated with or without 5 µg mL^−1^ HQNO and/or 0.005% oleic acid, and fluorescence was quantified. Each individual data bar corresponds to the average of the biological replicates (*n* = 3), and standard deviations are shown, but the data points sometimes obscure them. The data in the panels were analyzed using two-way analysis of variance, and *ad hoc* Tukey tests were conducted for pairwise comparisons. The * denotes a *P*-value ≤0.05, *** ≤0.001, and **** ≤0.0001, and ns denotes no significant difference. BDL denotes below the detectable limit of the fluorimeter.

The activity of SaeRS decreases as free fatty acids accumulate endogenously or when supplemented exogenously (35–37). We tested the hypothesis that respiratory inhibition by HQNO increases fatty acid levels. We quantified cell-associated free fatty acids in WT and Δ*cydA qoxB::Tn* strains after culture with or without HQNO. The concentration of total fatty acids was increased in the Δ*cydA qoxB::Tn* strain when compared to the WT. The levels of free fatty acids increased in the WT strain upon challenge with HQNO, while the respiration-deficient strain showed no significant difference in total free fatty acid concentrations (Fig. 5A). Importantly, the free fatty acid levels of the HQNO-treated WT phenocopied the levels seen in the Δ*cydA qoxB::Tn* strain.

Fatty acid-free BSA has a high affinity for fatty acids, and supplementing the growth medium with BSA lowers titers of fatty acids in the medium, shifting the equilibrium and resulting in lower intracellular fatty acid concentrations (66). The affinity of BSA for fatty acids increases as their carbon chain length increases (67). We cultured the WT strain containing the *saeP1* transcriptional reporter with HQNO and/or fatty acid-free BSA. As described above, the transcriptional activity of *saeP1* was decreased upon co-culture with HQNO; however, the addition of BSA directly to the medium significantly increased *saeP1* transcriptional activity compared to the cultures without BSA (Fig. 5B).

To ensure that the observed phenotypes were a result of BSA binding fatty acids and not HQNO, we combined HQNO, BSA, or BSA + HQNO with TSB and filtered the solutions through a 10 kDa molecular mass cutoff filter. We then added individual filtrates to the cultures of the WT containing the *saeP1* transcriptional reporter and quantified transcriptional output. The filtered HQNO significantly reduced *saeP1* transcriptional activity when compared with the vehicle control, whereas the filtered BSA solution did not (Fig. 5B). The filtrate generated from BSA + HQNO reduced *saeP1* transcriptional activity, suggesting BSA does not significantly bind HQNO under the conditions examined, which is consistent with a previous report (68).

Treatment of *S. aureus* with oleic acid decreases SaeRS output (49, 56). We examined whether HQNO treatment would interfere with the ability of oleic acid to interfere with SaeRS signaling. We challenged the WT containing the *saeP1* transcriptional reporter with HQNO, oleic acid, or both compounds. Treatment with HQNO or oleic acid decreased *saeP1* transcriptional activity (Fig. 5C). Treatment with both HQNO and oleic acid had an additive effect on the transcriptional activity of *saeP1*. Taken together, the data presented in Fig. 5 are consistent with the hypothesis that HQNO inhibits respiration, which results in fatty acid accumulation, decreasing the concentration of phosphorylated SaeR, and decreasing SaeRS-dependent transcriptional output.

## DISCUSSION

We and others have demonstrated that respiration status is tethered to the production of virulence factors in *S. aureus* and that SaeRS activity is modulated by respiratory status (38, 69, 70). Transcriptional profiling of *S. aureus* cells co-cultured with *P. aeruginosa* displayed reduced transcription of genes coding for several virulence factors, including SaeRS-regulated serine proteases and leukotoxins (19). Another study found that challenging *S. aureus* with HQNO resulted in decreased *hla* transcription (71). Herein, we tested the hypothesis that one or more *P. aeruginosa*-secreted secondary metabolites alter the transcriptional output of the *S. aureus* SaeRS TCRS, resulting in altered regulation of virulence factors.

Consistent with our hypothesis, adding *P. aeruginosa* cell-free conditioned culture medium to *S. aureus* decreased the transcriptional activities of two genes directly regulated by SaeRS. Importantly, both targets had no transcriptional activity in a Δ*saeRS* mutant, confirming that they require functional SaeRS for transcription (59, 60). Further analyses determined that HQNO was the primary metabolite responsible for the altered transcription of SaeRS-regulated genes. Previous studies demonstrated that HQNO can be found at concentrations up to 10 µg mL^−1^ in stationary-phase cultures of *P. aeruginosa,* and other studies have used this as a working concentration for their experiments (71–73). In a companion manuscript, we demonstrate that treatment of *S. aureus* with 5 µg mL^−1^ HQNO under our experimental conditions lowers the rate of dioxygen consumption and decreases the proton motive force, consistent with HQNO inhibiting respiration. *S. aureus* strains genetically modified to lack both NADH dehydrogenases or both terminal oxidases showed decreased SaeRS transcriptional output (Fig. 4). Schurig-Briccio et al. also reported that inactivation of both NADH dehydrogenases decreased expression of *hla* (39). Epistasis experiments herein demonstrated that HQNO treatment did not further decrease the transcriptional activity in these mutants defective in respiration. These data confirm that SaeRS transcriptional activity is impacted by respiratory status and demonstrate that active respiration is required for HQNO treatment to impact SaeRS signaling.

Adding exogenous fatty acids or accumulating cellularly produced free fatty acids not incorporated into phospholipids decreases SaeRS transcriptional output (35, 37, 49). An *S. aureus* strain lacking both NADH dehydrogenases (*ndh*) has decreased production of SaeRS-regulated virulence factors (35). Incubating the *ndh* mutant with fatty acid-free BSA, which binds fatty acids, decreased the concentration of fatty acids available to interact with the cells and increased SaeRS-regulatory output. Herein, we also demonstrate that treating cells with HQNO and BSA increased SaeRS-dependent transcriptional activity compared to treatment with HQNO alone. We also demonstrate that cell-associated fatty acids accumulate in a *S. aureus* mutant incapable of respiring and in cells treated with HQNO, confirming that decreased respiration results in fatty acid accumulation.

The biocide triclosan inhibits the function of the enoyl-acyl carrier protein reductase (FabI), which is required to synthesize fatty acids (74). We recently demonstrated that treating *S. aureus* with a growth-permissive concentration of triclosan increases SaeRS output (75). In contrast to what we discovered here, the growth of *S. aureus* with triclosan decreased the concentration of cell-associated fatty acids. The phenotypes associated with triclosan growth were nullified by including a fatty acid in the culture medium or by the introduction of a *fakA* mutation, which accumulates fatty acids.

This study raises several interesting questions that need to be addressed in the future. First, we need to determine why and how fatty acids accumulate in non-respiring *S. aureus* cultured in TSB. Our companion manuscript provides insight by demonstrating that growth with HQNO stimulates the staphylococcal respiratory regulator (SrrAB) and Rex transcriptional regulators, which regulate genes used for fermentation (76). Increased fermentation decreases carbon flux through the TCA cycle, which could cause carbon from acetyl-CoA to be shunted into fermentation pathways and fatty acid synthesis (77). Consistent with this, metabolomic analyses found that pyruvate, lactate, and acetyl-CoA accumulated upon treatment with HQNO (64). It is unknown how the fatty acids that are synthesized on the acyl-carrier protein are liberated, allowing for accumulation, but it is possibly due to an unidentified thioesterase. The data presented are consistent with a model where treatment of *S. aureus* with HQNO led to a decrease in the concentration of phosphorylated SaeR, but future biochemical studies will be necessary to directly quantify the titers of phosphorylated SaeR after treatment with fatty acids or HQNO to confirm whether and how fatty acids directly alter SaePQRS phosphatase or kinase activities.

**Fig 6:**
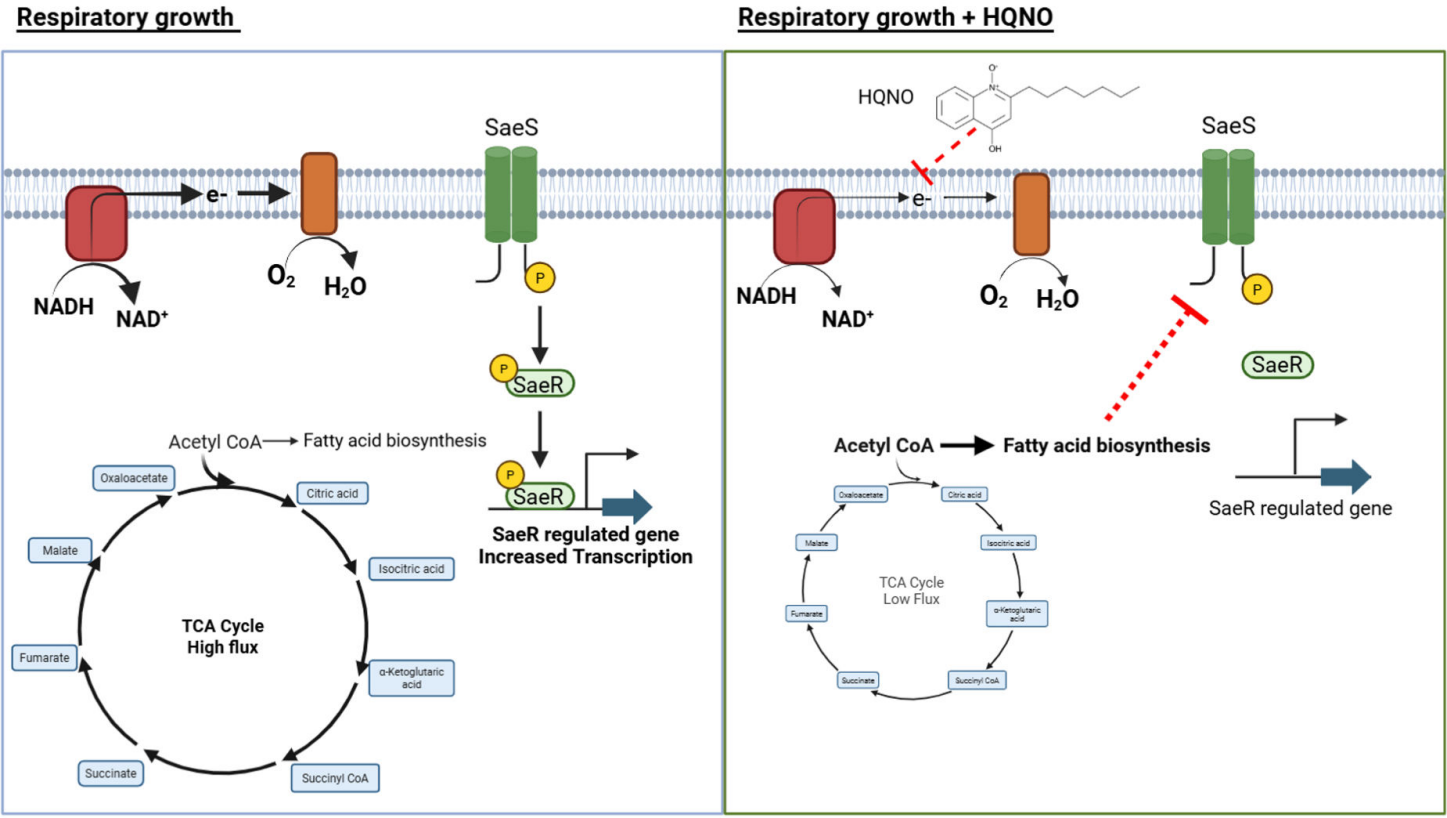
Working model for SaeRS-dependent regulatory changes upon HQNO challenge. Inhibition of respiration by HQNO decreases carbon flux into the TCA cycle. This increases the cellular concentration of acetyl-CoA, which is diverted into the fatty acid biosynthesis pathway. Fatty acids are liberated from the acyl-carrier protein by a currently unknown mechanism. The liberated fatty acids, either directly or indirectly, stimulate the SaePQS membrane complex, increasing phosphatase activity or decreasing kinase activity, resulting in decreased titers of phosphorylated cytosolic SaeR, lowering the affinity of SaeR for DNA and decreasing transcription of target genes.

The findings presented in this study, in combination with the findings in our companion manuscript (64), have led to a working model for the effect of *P. aeruginosa* on SaeRS signaling (Fig. 6). *P. aeruginosa* secretes HQNO, which inhibits respiration in *S. aureus*. Inhibition of respiration stimulates the SrrAB and Rex transcriptional regulators, decreasing the expression of genes that code for TCA cycle enzymes and increasing the expression of genes that function in fermentation. This causes carbon from glucose to accumulate as acetyl-CoA, part of which is converted into fatty acids. As fatty acids collect on the acyl-carrier protein, they are hydrolyzed off and accumulate. This accumulation increases the concentrations of fatty acids that can interact directly or indirectly with SaePQS, resulting in altered SaeS activity, decreased phosphorylated SaeR, and decreased transcription of SaeR-regulated genes.

The finding that *P. aeruginosa-*produced HQNO results in decreased *S. aureus* SaeRS-dependent transcription provides insight into the relationship between both organisms, which is relevant in infection settings such as the airways and lungs of cystic fibrosis patients. HQNO is thought to be secreted as a virulence factor against host cells and other bacteria, like *S. aureus*, during polymicrobial infections of bronchial epithelial cells (71, 78). Interestingly, co-culturing *S. aureus* with *P. aeruginosa* selects for *S. aureus* strains that have increased survival. These strains, known as small colony variants (SCVs), are characterized by a fermentative phenotype due to impaired respiration (79). Culture of *S. aureus* with a non-growth-permissive amount of pyocyanin, another *P. aeruginosa*-excreted secondary metabolite, resulted in the selection of SCVs (79, 80). Treatment of *S. aureus* with antibiotics that require the proton motive force for entry into the cell selects for SCVs (81). Approximately 25% of children with CF have been found to have *S. aureus* SCVs in their airways (82). SCV colonization has been associated with declining lung health and has been linked to increased colonization with *P. aeruginosa* as the CF infection progresses (82–84). *S. aureus* in the CF airway will likely have decreased access to dioxygen, but the selection for respiration-deficient strains suggests that some respiration is occurring. Therefore, inhibition of respiration and possibly the altered transcriptional regulation that occurs as a result could promote survival against *P. aeruginosa* and/or the human host.

Strains with mutations that increased or decreased SaeRS output have exhibited higher or lower virulence in infection models, respectively (30, 85, 86). The findings presented in this study raise the question of whether HQNO-dependent inhibition of respiration and the subsequent repression of SaeRS transcriptional output affect the ability of *S. aureus* to survive in the CF airway and lung. It also raises the question of whether decreasing SaeRS-dependent virulence factor production decreases the host response, promoting bacterial survival.
